# Roles of PI3K/AKT/GSK3 Pathway Involved in Psychiatric Illnesses

**DOI:** 10.3390/diseases7010022

**Published:** 2019-02-13

**Authors:** Satoru Matsuda, Yuka Ikeda, Mutsumi Murakami, Yukie Nakagawa, Ai Tsuji, Yasuko Kitagishi

**Affiliations:** Department of Food Science and Nutrition, Nara Women’s University, Kita-Uoya Nishimachi, Nara 630-8506, Japan; tyvufkxaq1226-218@outlook.jp (Y.I.); murakamimc@gmail.com (M.M.); yukiie0028@yahoo.co.jp (Y.N.); ai.tsuji0225@gmail.com (A.T.); y_kitagishi@live.jp (Y.K.)

**Keywords:** PI3K, AKT, GSK3, PTEN, cell signaling, schizophrenia, depression

## Abstract

Psychiatric illnesses may be qualified to the cellular impairments of the function for survival or death in neurons, which may consequently appear as abnormalities in the neuroplasticity. The molecular mechanism has not been well understood, however, it seems that PI3K, AKT, GSK3, and their downstream molecules have crucial roles in the pathogenesis. Through transducing cell surviving signal, the PI3K/AKT/GSK3 pathway may organize an intracellular central network for the action of the synaptic neuroplasticity. In addition, the pathways may also regulate cell proliferation, cell migration, and apoptosis. Several lines of evidence have supported a role for this signaling network underlying the development and treatment for psychiatric illnesses. Indeed, the discovery of molecular biochemical phenotypes would represent a breakthrough in the research for effective treatment. In this review, we summarize advances on the involvement of the PI3K/AKT/GSK3 pathways in cell signaling of neuronal cells. This study may provide novel insights on the mechanism of mental disorder involved in psychiatric illnesses and would open future opportunity for contributions suggesting new targets for diagnostic and/or therapeutic procedures.

## 1. Introduction

**Figure 1:**
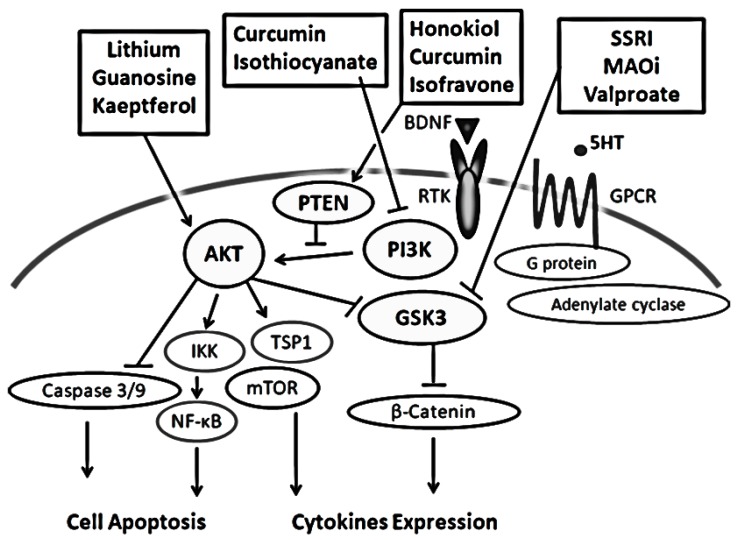
Potential antidepressants and several modulators linked to the predominant molecular targets on PI3K/AKT/GSK3 pathway are demonstrated. Arrowheads mean stimulation whereas hammerheads represent inhibition, suggesting implication of the PI3K/AKT/GSK3 modulators for the treatment of psychiatric illnesses. Note that some critical events have been omitted for clarity. SSRI: Selective Serotonin Reuptake Inhibitors, MAOi: Monoamine oxidase inhibitors, BDNF: Brain-derived neurotrophic factor, 5-HT: 5-hydroxytryptamine, serotonin.

Psychiatric illnesses are conditions for which the precise underlying reason remains unknown; however, roles of dysregulation of the signaling related to neurotransmitters, intracellular signal transduction, and neural development have been emphasized in the pathogenesis of these illnesses [1]. For example, it has been shown that dysfunction of dopamine D1 and/or D5 receptor signaling is implicated in schizophrenia [2], which is linked to the activation of PI3K/AKT signaling with the subsequent inactivation of GSK3. Activation of AKT brings an increase in the phosphorylation of GSK3. In addition, the regulation of PI3K/AKT/GSK3 signaling has also been implicated in the etiology of mood disorders and depression [3]. In fact, molecular AKT deletion evokes a change in behavior reflecting the psychiatric appearance reminiscent of schizophrenia, anxiety and depression [4]. Several G protein-coupled receptors (GPCRs) and receptor tyrosine kinases (RTKs) are involved in the activation of the PI3K-/AKT-mediated signaling [5,6]. Consequently, it has been indicated that selective activation of these receptors may be efficacious in treating some neuropsychiatric disorders (Figure 1). Therefore, PI3K/AKT/GSK3 signaling might be critical underlying psychiatric-related behaviors. Therapeutic effects of various psychiatric drugs are also mediated in part by their inhibition of the signaling (Figure 1). For example, the mood stabilizer lithium has been used for the treatment of schizophrenia, depression, and other mental illnesses, which has been shown to inhibit the GSK3 signaling [7]. However, the molecular mechanism through which lithium regulates the signaling activities has been poorly understood. Identification of key signaling pathways should be critical to uncover novel therapeutic targets and successful clinical interventions. Moreover, a better understanding of the intricate PI3K/AKT/GSK3 actions may allow the rational development both for the diagnosis and treatments with enhanced efficacy. 

## 2. Characterization of the PI3K/AKT/GSK3 Signaling Pathway in the Pathogenesis of Psychiatric Illnesses

Neuron survival mechanisms ordinarily depend on activation of phosphatidylinositol 3′-kinase (PI3K), which exists as a dimer comprising of a 110 kDa catalytic subunit (p110) and an 85 kDa (p85) regulatory subunit [8]. Downstream targets of cytoplasmic PI3K seem to affect cell apoptosis, cell metabolism, intracellular vesicles transport and so on [9]. The p110 subunit generates phosphoinositide PIP3 at the inner surface of plasma membrane, which supports to recruit the phosphoinositide dependent protein kinase-1 (PDK1) via its pleckstrin homology domain (PH). PDK1 phosphorylates then activate the AKT serine/threonine kinase. The production of PIP3s by PI3K at the plasma membrane is essential for the recruitment and activation of PH domain-containing proteins. This PI3K–PDK1–AKT signaling pathway is required for the survival of several neuronal cells [10]. The AKT kinase family is constituted by three isoforms termed AKT1, AKT2, and AKT3, which is implicated in a variety of cellular processes such as cell growth and survival. Although they are showing robust homologies, each isoform is encoded by a distinct gene [11]. The most abundant one is AKT1, which is ubiquitously expressed. AKT2 is expressed in insulin-responsive tissues including muscle, and AKT3 is considerably expressed in brain and testis [12]. Some lethality is observed in AKT1 KO mice and the surviving mice are extensively reduced in size [13]. AKT1 is phosphorylated by the PDK1 and by PDK2 [14]. AKT2 is important for glucose metabolism. In addition, signs of anxiety and depressive-like behaviors have been reported in AKT2 KO mice [15]. AKT3 KO mice exhibit small brains [16], suggesting that AKT3 could be an important regulator of brain development. In addition, AKT3 might also play a pivotal role in human brain pathologies such as schizophrenia. It is remarkable that studies have identified AKT3 as a potential contributor to schizophrenia [17,18]. Actually, deletion of AKT3 increases susceptibility to develop symptoms related to the disease [18]. Therefore, all AKT signaling may contribute to the functioning of neural networks prevailing in the symptoms associated with psychiatric diseases. AKT localize mostly to the cytoplasm, but they can also translocate in the nucleus on an extracellular stimulation. Nuclear PIP3s may mediate a broad range of processes including DNA damage response and cell cycle regulation [19,20].

The tumor suppressor PTEN, phosphatase and tensin homolog on chromosome 10, is a dual-specificity phosphatase with protein phosphatase activity and lipid phosphatase activity. Cells that lack PTEN have constitutively high levels of PIP3 and could activate downstream PI3K/AKT [21]. Conversely, overexpression of PTEN might be related to the activation of the cell apoptosis which can be correlated with repression of PI3K/AKT signaling [21]. Accordingly, neuronal cell survival and/or cell death may be attributed in part to the variations in PTEN expression [21]. Inhibition of PTEN saves normal synaptic function and thereby cognition in animal models of Alzheimer’s disease [22]. Conversely, overexpression of PTEN exhibits synaptic depression that imitates psychological depression [23]. PTEN mutations have been described in several patients with autism spectrum disorders (ASDs) and macrocephaly [24]. AKT activation by downregulation of PTEN might be significant to keep its neuro-protective effects. 

The GSK3 family is composed of two isoenzymes termed GSK3α and GSK3β, which have been initially recognized for the roles in insulin receptor signaling. GSK3 is constitutively active serine/threonine kinase in cells. The activity of GSK3 is positively regulated by phosphorylation on tyrosine residues (Thy 279 for GSK3α and Thy 216 for GSK3β) [25] and negatively regulated by serine phosphorylation (Ser 21 for GSK3α and Ser 9 for GSK3β) [26]. A known negative regulator of GSK3 is a member of AKT. Phosphorylation of AKT on Thr 308 has been shown to be essential and sufficient for the regulation of GSK3 by AKT [14]. Levels of phosphorylated GSK3 are strongly decreased in the hippocampus of AKT3 KO mice. Circadian rhythm in phosphorylation of GSK3β, but not GSK3α, in hippocampal neurons has been reported [27]. Overall, the effect of depletion in brain GSK3 activity is a reduction in anxiety level that is associated with an increase in the beginning of social interaction.

## 3. Some Diagnostic Clues for Psychiatric Illnesses at the Molecules Involved in PI3K/AKT/GSK3 Pathway

In general, high mortality of diseases is mostly due to a lack of effective treatments and efficient markers for early diagnosis. The PI3K/AKT/GSK3 signaling cascade may be a center for psychiatric illnesses. If alteration of the signaling activities in brain neurons should be also detected in peripheral blood lymphocytes of illnesses patients, it could work for efficient diagnosis of the illnesses. In human lymphocytes, levels of PI3K subunit p110 have been impaired in patients with schizophrenia [28]. On the other hand, SNP within the PI3K subunit p85-gene is associated with a risk of alcohol drinking behavior [29]. It is remarkable that AKT1 has been originally identified as a possible susceptibility gene for schizophrenia [30]. AKT2 deletion has also been associated with anxiety- and depression-like behaviors [15,31]. In addition, injured AKT3 genes have been associated with psychiatric illnesses including schizophrenia. In consistent with this, AKT3 KO mice have demonstrated a phenotype reminiscent of depression and schizophrenia. In addition, characters of animals with the AKT3 deletion have shown microcephaly [32], whereas high-AKT3 activities are associated with macrencephaly [33]. Psychiatric behaviors might be induced from the reduced brain volume brought by reduced AKT activity. Remarkably, alteration of the GSK3 activity has also been recognized as a schizophrenia risk factor [34]. Tissue samples from post mortem patients with schizophrenia have exhibited considerable reductions of the phosphorylated AKT levels in neurons [35]. Furthermore, AKT activity has also been reduced in some brain regions of major depression patients [36]. Phosphorylated AKT levels have been shown as decreased in a depression animal model [37]. Activation of the dopamine receptor 2 (D2R) has been revealed to stimulate the inactivation of the AKT by the protein phosphatase 2A [38], suggesting that GPCR activation could regulate the AKT in response to extracellular signals. An endogenous neuro-steroid in the central nervous system, pregnenolone, normalizes schizophrenia-like behaviors via the AKT signaling [39]. Thus, PI3K/AKT/GSK3 signaling may play a critical role in psychiatric appearances.

GSK3 knock-in mice have revealed high susceptibility to depressive behaviors [40]. In addition, impaired GSK3 activity has been documented to play a role in psychiatric conditions [41]. High activity of GSK3 has been found in bipolar disorder with circadian dysregulation [42]. Some anxiety and depressive behaviors have been revealed to be associated with lower brain levels of the phosphorylated GSK3 [43]. In particular, GSK3β is a common target of several psychoactive drugs. On the other hand, mutations in the PTEN are also extremely related with autism and macrocephaly. In addition, loss of PTEN may lead to an overall loss in interneurons [44]. Some mutations of the PTEN gene may disrupt the normally balanced nuclear-cytoplasmic localization of the PTEN phosphatase, which causes inappropriate behavior, a profile reminiscent of ASD, in animal models [45].

Noncoding 20–25-nucleotide-long RNAs termed microRNAs (miRNAs) have biological functions such as cellular proliferation and apoptosis, which could modulate gene expression by miRNA-induced silencing. The potential application of miRNAs has been considered as an early detection biomarker for illnesses. Genome studies have revealed genetic variants adjoining a miR-137 region may contribute to schizophrenia risk [46], suggesting that dysregulation of the miR-137 may contribute to schizophrenia pathogenesis by modifying neurodevelopmental signaling [47]. AKT signaling pathway has been shown involved in the miR-137 pathway [48]. In addition, miR-144-3p seems to be a viable target for posttraumatic stress disorder and related disorders [49]. Furthermore, several miRNAs including miR-16, miR-182, miR-223, and miR-451 have shown potential biomarkers in the condition of depression [50,51]. The miRNA-mediated modification of gene expression has been in part revealed via the PI3K/AKT/GSK3 signaling [51]. In relation to those, miRNAs let-7b and let-7c are also potential biomarkers of treatment-resistant depression, which regulates the expression of several genes in the PI3K/AKT/GSK3 pathway [52].

## 4. Some Diets With Phytocompounds May Contribute to the Neuro-protection in the Psychiatric Diseases via the Modulation of PI3K/AKT/GSK3 Signaling

Mood stabilizers, antidepressants and antipsychotics all may upturn the PI3K/AKT/GSK3 signaling [53], resulting in the regulation of GSK3 [53]. For example, schisandrin has an antidepressant-like effect, which possibly mediated partly by adjusting the PI3K/AKT/GSK3 signaling [54]. Furthermore, lithium administration repairs the phosphorylated GSK3 levels and improves anxiety-related behavior, which regulates neuronal cell death and increases neurogenesis. So, antidepressants such as lithium may protect the functional neuroplasticity in neurons associated with the depression. Lithium is an inhibitor of magnesium and can competitively inhibit Mg2+-ATP-dependent catalytic activity of the GSK3. The antipsychotic drug haloperidol has been linked to the inhibition of the AKT signaling [55]. In consistent with this, methamphetamine could induce psychosis, which is associated with highly increased expression of AKT [56]. 

As the efficacy of pharmacological treatments has been imperfect and had unexpected side effects, psychiatric illnesses represent a significant public health problem. A number of preventive factors have been suggested by epidemiological research including lifestyle factors such as diet and physical exercise. Among them, dietary choices could play certain roles in the neuroprotection. In addition, some involvements of food ingredients might be promising in the prevention of brain dysfunction by modifying the PI3K/AKT/GSK3 signaling. For example, an active ingredient derived from the root of *Curcuma longa* used as culinary turmeric, curcumin, can improve synaptic plasticity and enhance memory abilities [57]. The neuroprotection of the curcumin might be mediated through the modification of PI3K/AKT/GSK3 signaling [58]. In addition, the curcumin prevents oxidative stress via the regulation of the PI3K/AKT/GSK3 signaling [59]. Several plants or fruits may also be encouraging. A flavanone found in a variety of plants, Liquiritigenin, might be useful for the treatment of major depression via the modification of PI3K/AKT/GSK3 signaling [60]. Kaempferol is also a flavonol existing in several plants with grapefruit and some edible berries, which could protect some neurons [61]. On the contrary, exposure to high fat interrupts brain dopamine networks through the declines of striatal AKT activity [62]. Serine phosphorylation of GSK3 has been reduced in experimental animal models fed with a diet containing abundant stearic and/or palmitic acid [63]. Fatty acids such as DHA and EPA, omega-3 fatty acids found in fish, may also modulate neurotransmitters and support neurogenesis [64]. Supplementation with the omega-3 fatty acids has improved symptoms of depression patients [65]. In addition, consumption of the omega-3 fatty acids has mood-stimulating effects in patients [66]. Fatty acids supplementation has also shown positive effects in schizophrenia [67]. Anti-depressant effects of the omega-3 fatty acids could be induced by upregulation of the PI3K/AKT/GSK3 pathway [68].

Neuroprotection by inhibiting PTEN tumor suppressor with food ingredients has been described by activating the PI3K/AKT/GSK3 signaling in neuronal cells. For example, a traditional medicinal herb ingredient, Icariin, inhibits the PTEN expression following the AKT activation [69]. In addition, an active component isolated from Chinese traditional herb magnolia, Honokiol, may be able to attenuate PI3K/AKT/GSK3 signaling by upregulation of the PTEN expression [70]. Generally, dietary exposure to phytoestrogens including soy isoflavones may result in an increase of PTEN expression. Genistein and/or quercetin have an outcome to upregulate PTEN transcription following suppression of the PI3K/AKT/GSK3 pathway. Dietary intake of a phytochemical found in some vegetables such as indole-3-carbinol also upregulates PTEN expression [71]. As the PTEN expression is induced by the activated PPARs, this may also suggest a potential therapeutic quality for the management of PI3K/AKT/GSK3-related diseases. A wide variety of compounds have been identified as PPAR ligands including omega-3 fatty acids [72], which have a valuable effect on the risk factors for metabolic diseases. Linoleic acid could also bind PPARδ very well [73]. Fish oil diets have significantly increased the level of PTEN expression to reduce PI3K/AKT/GSK3 signaling [74]. These might be distinguished as a rational basis for the development of dietary treatments for psychiatric illnesses. 

## 5. So What Next in Perspectives?

**Figure 2:**
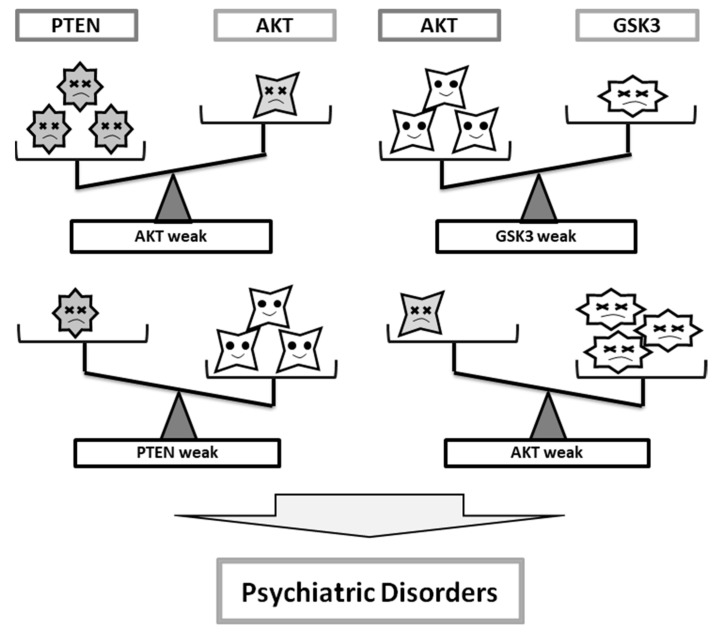
Imbalance of the activity among PTEN, AKT, and GSK3 may contribute to the pathogenesis of psychiatric disorders.

**Figure 3:**
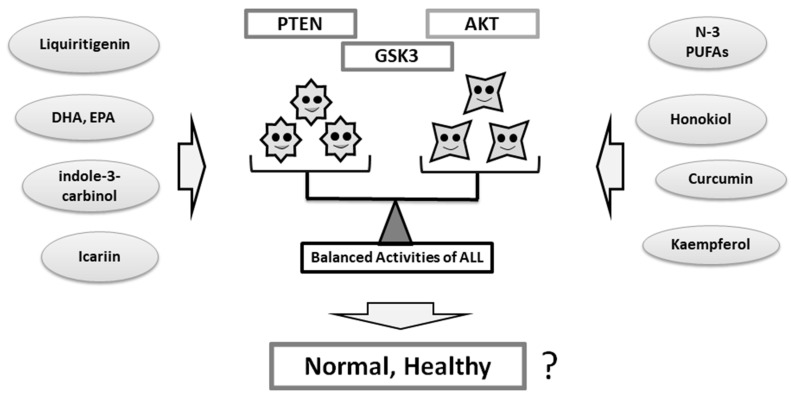
The balance of PTEN, AKT, and GSK3 in the meaning of their functions may be important for individual psychiatric health. Several food and/or dietary components may provide improved balance of the signaling via the modulation of the functional activities.

Lifestyle interventions such as dietary education could be promising and cost-effective for people with psychiatric illnesses. However, despite those experimental observations, the precise mechanisms for food ingredients remain elusive for clinical uses. A number of studies have examined the association between diet and behavioral states, but the findings have been inadequate. In addition, the relation between nutrient intake and neuroprotection activity is intricate. Whereas many questions remain to be answered about the roles of PI3K/AKT/GSK3 signaling in psychiatric disorders, it is possible that the PI3K/AKT/GSK3 signaling of neuronal populations in a certain brain area could be associated with distinct behavioral outcomes. It seems that both activation and inhibition of those molecules, if they drive one-sidedly, may not contribute to the improvement of the neuronal disorders (Figure 2). It seems that back-and-forth activation and/or inhibition for the appropriate balance may be very important (Figure 3). In other words, the functional balance of the activity of kinases may be essential. Strategies for seeking efficient therapy to determine whether it is related to the improved brain function and the preservation of brain health should develop the observation in key pathways required for brain homeostasis. Several nutriment and/or dietary components may contribute to the balance via the modulation of the kinase activities (Figure 3). Future findings might be translated into new dietary managements for the treatment of the disorders. Indeed, the PI3K/AKT/GSK3 pathway seems to be critical for the maintenance in brain neurons. Additionally, it seems important to exploit the potential benefits of optimal treatment and/or combination with PI3K/AKT chemical modulators. More understanding of the precise intracellular mechanisms downstream of PI3K/AKT/GSK3 changes in psychiatric illnesses could provide novel insights into the development of new therapeutic approaches with greater efficacy.

## 6. Conclusions

In conclusion, progress in understanding molecular key targets in order to develop new therapeutics for psychiatric illnesses would undoubtedly benefit this research field. The involvement of PI3K/AKT/GSK3 signaling in schizophrenia and mood disorders is highly relevant. Some diets may contribute to the neuro-protection in psychiatric illnesses via the modulation of the intracellular neuronal signaling. Therefore, improving treatments for human neuropsychiatric disorders by dietary approach is particularly challenging. Further investigation on the neuronal signaling could lead to a better understanding of the molecular basis implicated in neuropsychiatric illnesses.

## Abbreviations

ASD	Autism Spectrum Disorder
DHA	docosahexaenonic acids
EPA	eicosopentaenoic acid
GSK3	Glycogen synthase kinase 3
5-HT	5-hydroxytryptamine, serotonin
mTOR	mammalian target of rapamycin
PIP3	phosphatidylinositol 3,4,5-triphosphate
PI3K	phosphatidylinositol-3 kinase
PPARγ	Peroxisome Proliferator-Activated Receptor γ
PTEN	Phosphatase and tensin homolog on chromosome 10
ROS	reactive oxygen species
SSRIs	selective serotonin reuptake inhibitors
